# Genome-wide analysis of oxylipins and oxylipin profiles in a pediatric population

**DOI:** 10.3389/fnut.2023.1040993

**Published:** 2023-03-28

**Authors:** Teresa Buckner, Randi K. Johnson, Lauren A. Vanderlinden, Patrick M. Carry, Alex Romero, Suna Onengut-Gumuscu, Wei-Min Chen, Soojeong Kim, Oliver Fiehn, Brigitte I. Frohnert, Tessa Crume, Wei Perng, Katerina Kechris, Marian Rewers, Jill M. Norris

**Affiliations:** ^1^Department of Epidemiology, Colorado School of Public Health, Anschutz Medical Campus, Aurora, CO, United States; ^2^Department of Kinesiology, Nutrition, and Dietetics, University of Northern Colorado, Greeley, CO, United States; ^3^Department of Biomedical Informatics, CU School of Medicine, Anschutz Medical Campus, Aurora, CO, United States; ^4^Colorado Program for Musculoskeletal Research, Department of Orthopedics, CU School of Medicine, Anschutz Medical Campus, Aurora, CO, United States; ^5^Center for Public Health Genomics, University of Virginia, Charlottesville, VA, United States; ^6^Department of Health Administration, Dongseo University, Busan, Republic of Korea; ^7^NIH-West Coast Metabolomics Center, University of California-Davis, Davis, CA, United States; ^8^The Barbara Davis Center for Diabetes, CU School of Medicine, Anschutz Medical Campus, Aurora, CO, United States; ^9^Department of Biostatistics and Informatics, Colorado School of Public Health, Anschutz Medical Campus, Aurora, CO, United States

**Keywords:** oxylipin, inflammation, pediatric, genome-wide association study, polyunsaturated fatty acids, lipid mediators

## Abstract

**Background:**

Oxylipins are inflammatory biomarkers derived from omega-3 and-6 fatty acids implicated in inflammatory diseases but have not been studied in a genome-wide association study (GWAS). The aim of this study was to identify genetic loci associated with oxylipins and oxylipin profiles to identify biologic pathways and therapeutic targets for oxylipins.

**Methods:**

We conducted a GWAS of plasma oxylipins in 316 participants in the Diabetes Autoimmunity Study in the Young (DAISY). DNA samples were genotyped using the TEDDY-T1D Exome array, and additional variants were imputed using the Trans-Omics for Precision Medicine (TOPMed) multi-ancestry reference panel. Principal components analysis of 36 plasma oxylipins was used to capture oxylipin profiles. PC1 represented linoleic acid (LA)- and alpha-linolenic acid (ALA)-related oxylipins, and PC2 represented arachidonic acid (ARA)-related oxylipins. Oxylipin PC1, PC2, and the top five loading oxylipins from each PC were used as outcomes in the GWAS (genome-wide significance: *p* < 5×10^−8^).

**Results:**

The SNP rs143070873 was associated with (p < 5×10^−8^) the LA-related oxylipin 9-HODE, and rs6444933 (downstream of *CLDN11*) was associated with the LA-related oxylipin 13 S-HODE. A locus between *MIR1302-7* and *LOC100131146*, rs10118380 and an intronic variant in *TRPM3* were associated with the ARA-related oxylipin 11-HETE. These loci are involved in inflammatory signaling cascades and interact with *PLA2*, an initial step to oxylipin biosynthesis.

**Conclusion:**

Genetic loci involved in inflammation and oxylipin metabolism are associated with oxylipin levels.

## 1. Introduction

Oxylipins are oxygenated lipid mediators derived from omega-6 (n6) and omega-3 (n3) fatty acids (FA), which are polyunsaturated fatty acids (PUFA). Generally, n6 fatty acids (FA) demonstrate pro-inflammatory properties, and n6 FA demonstrate anti-inflammatory properties (1, 2). The inflammatory properties of n6 and n3 FA are partially related to the actions of oxylipins. Oxylipin biosynthesis begins with cleavage of PUFA from phospholipids in the cell membrane *via* phospholipase A2 (PLA2) (3). Biosynthetic enzymes, including cyclooxygenase (COX), lipooxygenase (LOX), and cytochrome P450 (CYP450) synthesize oxylipins from these cleaved PUFAs. Oxylipins synthesized from the COX enzyme bind to G-protein-coupled receptors, PPAR-g, and other receptors (4). The six LOX enzymes (5-LOX, 12-LOX, 12/15-LOX, 15-LOX type 2, 12(R)-LOX, and epidermal LOX (5)) convert PUFAs to hydroxyl FAs, that also bind to G-protein-coupled receptors and other cellular receptors to regulate inflammation (4). The CYP450 enzymes also synthesize oxylipins, which play a role in vasodilation and immune modulation, including reduction in monocytes and inflammation (4, 6). Importantly, n6 FA compete with n3 FA for the same oxylipin biosynthetic enzymes (4, 7).

Oxylipins are inflammatory biomarkers and have thus been investigated in inflammatory conditions, such as asthma (8), lung inflammation (9, 10), chronic obstructive pulmonary disease (9, 10), obesity (11), and type 2 diabetes (12). Generally, oxylipins derived from n3 FA have anti-inflammatory, anti-proliferative, and pro-resolving properties (4). In contrast, most oxylipins derived from n6 FA are inflammatory, vasoconstrictive, and proliferative. However, some oxylipins derived from the n6 FA, linoleic acid (LA), exhibit anti-inflammatory effects (4). We investigated the association between oxylipins and T1D and used a principal component analysis (PCA) to capture the interconnected networks of multiple oxylipins (13). We found that PC1, representing LA-and ALA-related oxylipins and PC2, representing ARA-related oxylipins were associated with T1D. Although oxylipins are highly relevant to many inflammatory conditions, there has not yet been a genome-wide association study (GWAS) conducted with oxylipins, which would shed light on genetic factors and biological pathways potentially contributing to oxylipins and disease.

There are well-characterized genetic determinants of the precursor PUFA. The *FADS* genes (14, 15) encode the fatty acid desaturase enzyme, involved in desaturation of LA and alpha-linolenic acid (ALA), which are rate-limiting steps for the production of PUFA (14, 16–19) and are known determinants of PUFA. Similarly, *ELOVL* genes elongate PUFAs, and in a GWAS, SNPs in the *ELOVL2* gene region were associated with higher eicosapentaenoic acid (EPA) and docosapentaenoic acid (DPA), and lower docosahexaenoic acid (DHA) levels (14)*. LPCAT3* facilitates the transfer of lipids between glycerophospholipids and is another identified locus that may account for 4–8% of the variation in LA levels (20). Wolf et al. utilized a PCA of summary statistics from GWAS of n3 and n6 FA. This study replicated findings in the *FADS1, ELOVL2*, and *LPCAT3* genes and identified novel loci in the *PTPRM* and *AGPAT4* genes (21), and demonstrated that a PCA of n3 and n6 is a valid method for replication of previous findings as well as discovery of novel loci.

Although there has not been a GWAS of oxylipins, there are reports of targeted analyses based on gene regions associated with PUFA and oxylipin biosynthetic enzymes. For example, a SNP in the *FADS* gene, rs174537, was associated with levels of the oxylipins LTB4 and 5-HETE (15). The *ALOX5* (5, 12), *ALOX12* (22), *PTGS2* (22), and *EPHX2* (23) genes have been investigated because these genes encode for oxylipin biosynthetic enzymes. Variants in the promoter region for *ALOX5* were associated with lower leukotriene production in eosinophils from children with asthma (24), and variants in this region also altered arachidonic (ARA)-, EPA-, and DHA-derived oxylipins in monocytes in healthy adults (25). Variants in the *ALOX12* region were associated with increased urinary levels of the oxylipin 12(S)-HETE (26). Bermingham et al. found that both genetic and environmental predictors contribute to variation in oxylipins (27). Genetic influences contributed the most to the variation in oxylipins synthesized by 12-LOX, including 12-HETE and 12-HEPE. We found that dietary intake and red blood cell levels of the precursor PUFAs are associated with their respective oxylipins, and that many oxylipins change with age (28), but it is important to identify genetic factors that additionally contribute to the variation in oxylipins.

Although targeted analyses have identified genetic regions related to biosynthetic enzymes that may contribute to oxylipin levels, it is important to determine other regions of the genome that also contribute to the variation in oxylipin levels. Because of the pivotal role that oxylipins play in inflammatory conditions, identifying genetic loci associated with oxylipin levels may illuminate genetic influences for these oxylipin-related conditions. PCA of quantitative traits has been used in GWAS to identify novel loci and replicate previous loci for pleiotropic traits (21, 29, 30), so we assessed both oxylipin PCs and individual oxylipins. We used a genome-wide approach to investigate genetic predictors of oxylipin profiles captured through principal components (PCs) and individual oxylipins that characterize the PCs, in children at risk of T1D.

## 2. Materials and methods

### 2.1. Study population and study selection

The study population was drawn from the Diabetes Autoimmunity Study in the Young (DAISY), a longitudinal cohort study in Denver, CO following 2,547 children at risk of T1D. Children were recruited into the DAISY cohort if they had a first degree relative with T1D or had a high-risk human leukocyte antigen (HLA) genotype. The high-risk T1D genotype (HLA) was defined as DRB1*04, DQB1*0302/DRB1*0301, DQB1*0201 (DR3/4 DQ8) (31). Study visits were conducted at 9, 15, and 24 months, then annually. Radio-binding immunoassays were used to test subjects for serum autoantibodies to GAD65, IAA, IA-2, and ZnT8 as described previously (32). Islet autoimmunity (IA) was defined as testing positive for at least one islet autoantibody on two or more consecutive visits or testing positive for islet autoantibodies followed by diagnosis with T1D at the next visit (within a year) using ADA criteria (33).

A nested case–control study of IA cases and controls was conducted within DAISY (13). Controls, who were autoantibody- and T1D-free, were frequency matched to the cases on age at IA seroconversion of the case, race-ethnicity, and sample availability. A maximum of four study visits per child were selected to be included in the nested case–control study. After blood draw, plasma was separated immediately and snap-frozen in liquid nitrogen, then stored at −70°C. Blood draws were non-fasting. We selected all participants with oxylipins and genetic variants measured, which included 339 participants from the IA nested case–control study. We removed related individuals by randomly selecting one individual from each group of siblings, leading to a final sample size of *n* = 316.

### 2.2. Measurement of oxylipins

As previously described, oxylipins were quantified in plasma (34) in the IA nested case–control study. Extracted oxylipins were separated and quantified using a Waters i-Class Acquity UHPLC system coupled to a Sciex 6,500+ QTRAP mass spectrometer in negative ionization mode. Oxylipins were quantified by targeted, retention time specific, Multiple Reaction Monitoring (MRM) ion transitions. A total of 78 oxylipins were targeted, and targets that appeared in any sample above the signal-to-noise ratio of 3:1 were quantified. This resulted in quantified values for 27 n6-related oxylipins, including 17 ARA-related and 10 LA-related oxylipins. There were 12 n3-related oxylipins measured, which included 6 ALA-related, 4 DHA-related, and 2 EPA-related oxylipins. Two oxylipins (10-Nitrooleate and 9–10-e-DiHO) were quantified but not included in analyses because the precursor FA is oleic acid. Oxylipins measured in a sample below the limit of quantitation (LOQ), defined as signal-to-noise ratio below 3:1, were converted to 10% of the LOQ. Full names, corresponding enzymes, and precursor FA are listed in Supplementary Table S1. Relationships between precursor FA and oxylipins are illustrated in Supplementary Figure S1. Measuring both n6- and n3-derived oxylipins captures pro-inflammatory and anti-inflammatory signals. Oxylipins were Box-Cox transformed for normality before analysis.

### 2.3. Summary measure of oxylipins and oxylipin profiles

As described previously, we created a summary measure of the plasma oxylipins and have two profiles of interest that were previously associated with T1D (13). In brief, we calculated an age-adjusted average oxylipin measure by implementing a linear mixed model with age as the predictor and Box-Cox transformed oxylipin as the outcome, with a random intercept and unstructured covariance structure (13). Models did not converge for three ARA-related oxylipins (PGE2, 6-trans-LTB4 and PGD2) and were not included in subsequent analyses. Next, because oxylipins share precursor FA and compete for biosynthetic enzymes, we used PCA of the oxylipin intercepts to create oxylipin profiles to capture the intercorrelations among multiple oxylipins (13) and identified 2 profiles of interest (PC1 and PC2), which were subsequently tested with risk of T1D as reported (13). Based on the loading values from the data driven approach of PCA, we found that oxylipin PC1 represented largely LA- and ALA-related oxylipins, and oxylipin PC2 represented largely ARA-related oxylipins (Supplementary Table S2).

### 2.4. Selection of outcomes

Previously identified oxylipin profiles that were found to explain a large proportion of variance of the oxylipin panel in our cohort and are associated with T1D (13) were selected as outcomes in GWAS. In addition, we selected the top 5 oxylipins with the largest loading values for each profile of interest, to further investigate genetic associations of individual oxylipins, capture specific metabolic pathways, and aid in interpretation of the oxylipin PCs. For oxylipin PC1, 12,13-DiHOME, 13 S-HODE, 9-HODE, 9,10-DiHOME, and 9,10-DiHODE were selected and for oxylipin PC2, 11-HETE, 5-HETE, 12 S-HETE, 11,12-DiHETrE, 14,15-DiHETrE were selected (Supplementary Table S2).

### 2.5. Genotyping, imputation, and quality control

DNA samples were genotyped on the custom designed TEDDY-T1D Exome array at the University of Virginia (UVA) Genome Sciences Laboratory following the manufacturer’s protocol (Illumina). The Illumina GeneTrain2 algorithm was used to generate genotype clusters. Stringent SNP- and sample-level quality-control filtering and data cleaning were performed to ensure high-quality genotypes were used for the imputation step. The following variant filters were applied: (1) For sample filtering, samples with a genotype call rate < 0.95 were excluded. To identify potential sample mix-ups we used X-chromosome heterozygosity and Y-chromosome missingness to identify and exclude participants for whom reported sex did not match genotype inferred sex (35). We used KING to identify cryptic relationships (35) and (2) For SNP filtering, SNPs were removed if the SNP was identified as being monomorphic, or deviation from Hardy–Weinberg equilibrium test (*p* < 1×1^−20^ at the MHC region or *p* < 1×10^−6^ otherwise). A total of 445,541 SNPs passed quality control metrics.

Genotypes at additional variants were imputed using the Trans-Omics for Precision Medicine (TOPMed) multi-ancestry reference panel (version r2) resulting in a total of 292,174,934 variants (36–38). Rsq was calculated as the estimated squared correlation of the imputed genotype and the true (unobserved) genotype. We selected SNPs with a minor allele frequency (MAF) ≥0.05 and Rsq ≥ 0.7, which led to the inclusion of 6,196,887 variants for GWAS. Chromosomal position is based on NCBI genome build hg38.

### 2.6. Statistical analysis

Baseline characteristics were described for the study population. Genome wide association analyses (GWAS) was run through PLINK v1.9 (39), adjusting for ancestry and sex. Multiple linear regression was used for the GWAS, with oxylipin PC1, PC2, and the 10 selected oxylipins (12,13-DiHOME, 13 S-HODE, 9-HODE, 9,10-DiHOME, and 9,10-DiHODE from oxylipin PC1 and 11-HETE, 5-HETE, 12 S-HETE, 11,12-DiHETrE, 14,15-DiHETrE from oxylipin PC2). To estimate population ancestry, PCA was performed on the SNPs meeting the filtering criteria. The PCA projection analysis was performed in the reference population (i.e., the controls). We selected 3 principal components (PC1-3) based on the Scree plot to adjust for ancestry (Supplementary Figure S2). Based on value of p histograms (Supplementary Figure S3), we determined the results were not overinflated. A value of *p*<5 × 10^−5^ was considered as suggestive evidence of association, and a value of *p*<5 × 10^−8^ was considered as genome-wide significant. Manhattan plots were created for all GWAS. LocusZoom plots were used to investigate regions that met suggestive significance for oxylipin PC1 and PC2 and regions that met genome-wide significance for individual oxylipins (40). We selected any SNP meeting these criteria for individual oxylipins (genome-wide significance) and the two lead SNPs for oxylipin PC1 and PC2 (suggestive significance) and combined into non-overlapping windows of ±500 KB for regional plots. Lead SNP is indicated by the diamond shape, circles indicate a positive beta estimate value, and triangles indicate a negative beta estimate value.

## 3. Results

### 3.1. Participant description

This study included 316 participants from the DAISY cohort, of whom 49% were female, and 28% had a high-risk HLA genotype. 80% of the participants reported non-Hispanic White (NHW) ethnicity, and age at oxylipin measurement ranged from 8 months-22 years. Additional participant characteristics are described in Table 1.

**Table 1:** Participant characteristics (*n* = 316).

Characteristic	*n* (%)
Sex (Female)	155 (49.05%)
Non-Hispanic white ethnicity (yes)	253 (80.06%)
HLA-DR3/4 genotype (yes)	90 (28.48%)
First degree relative with T1D (yes)	175 (55.38%)
Islet Autoantibody Positive	157 (49.68%)

High-risk T1D genotype (HLA) was defined as DRB1^*^04, DQB1^*^0302/DRB1^*^0301, DQB1^*^0201 (DR3/4 DQ8).

### 3.2. Oxylipin PC1

**Figure 1:**
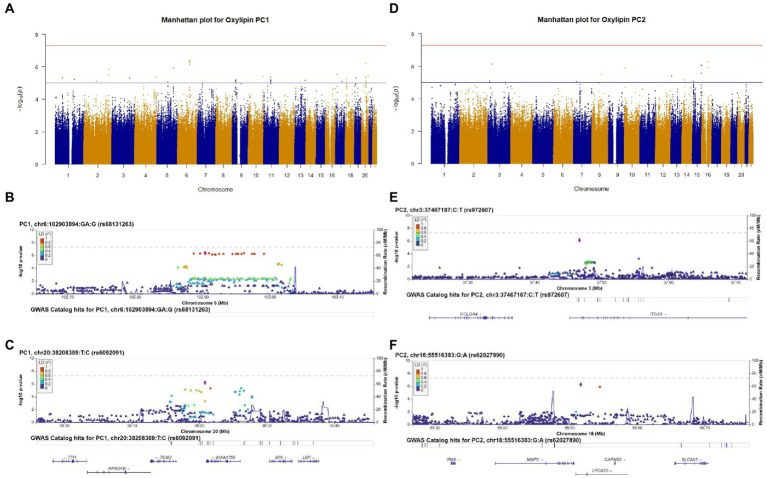
Genome-wide association study (GWAS) results of oxylipin PC1 **(A)** and oxylipin PC2 **(D)**, adjusted for sex and ancestry. Suggestive significance (value of *p*<5 × 10^−5^) is noted by the blue line, and genome-wide significance (value of p<5 × 10^−8^) is noted by the red line. We selected the two lead SNPs from oxylipin PC1 and PC2 that met suggestive significance for regional plots. Regional plots are for oxylipin PC1 **(B,C)** and PC2 **(E,F)**. Chromosome and physical location (Mb) are on the X-axis. Negative base ten logarithm of the value of ps is on the left Y-axis, and recombination activity (cM/Mb) is shown on the right Y-axis as a blue line. Positions, recombination rates, and gene annotations are according to NCBI’s build 38 (hg 19) and the 1,000 Genomes Project Phase 3 multiethnic data set. LD is calculated for a European population. Plots display +/−500,000BP of the selected SNP.

We conducted a GWAS of oxylipin PC1 to identify genetic predictors of an oxylipin profile characterized by LA- and ALA-related oxylipins. No SNPs met genome-wide significance (value of *p* <5 × 10^−8^) for oxylipin PC1, and 290 SNPs met suggestive significance (value of p <5 × 10^−5^), after adjusting for sex and ancestry. The Manhattan plot (Figure 1A) illustrates the results of the GWAS. The lead SNP for oxylipin PC1 was rs68131263 (Figure 1B; Table 2), an indel located in an intergenic region between *TARDBPP5* and *LOC105377916* (beta estimate: 0.461, *p* = 4.06 × 10^−7^). The second lead significant SNP for oxylipin PC1 was rs6092091, located in an intergenic region between the *TGM2* and *KIAA1755* genes (Figure 1C). The C allele was positively associated with oxylipin PC1 (beta estimate 0.488, *p* = 6.17 × 10^−7^). SNPs flanking rs6092091 in high LD exhibit similar levels of significance and directionality.

**Table 2:** Top SNPs Associated with Oxylipin PC1 and PC2.

SNP ID	rs number	Gene location	*β*	*p*	MAF	Rsq
*Lead SNPs for oxylipin PC1 meeting suggestive significance*						
chr6:102903894:GA:G	rs68131263	Between *TARDBPP5* and *LOC105377916*	0.461	4.06 × 10^−7^	0.29	0.99
chr20:38208389:T:C	rs6092091	Between *TGM2* and *KIAA1755*	0.488	6.17 × 10^−7^	0.24	0.95
*Lead SNPs for oxylipin PC2 meeting suggestive significance*						
chr3:37467187:C:T	rs972607	*ITGA9*: Intron Variant	0.772	7.20 × 10^−7^	0.07	0.95
chr16:55516383:G:A	rs62027890	*LPCAT2*: Intron Variant	0.618	5.21 × 10^−7^	0.11	0.87


GWAS were adjusted for sex and ancestry PC1-PC3.

SNPs are identified as chromosome number: base pair location: reference allele: alternate allele.

All beta estimates are calculated with respect to the reference allele (i.e., a positive beta estimate implies that the reference allele is associated with higher average levels of the corresponding oxylipin).

Minor allele frequency (MAF).

Rsq was calculated as the estimated squared correlation of the imputed genotype and the true (unobserved) genotype.

Suggestive significance defined as p < 5 × 10^−5^.

### 3.3. Oxylipin PC1 individual oxylipins

**Figure 2:**
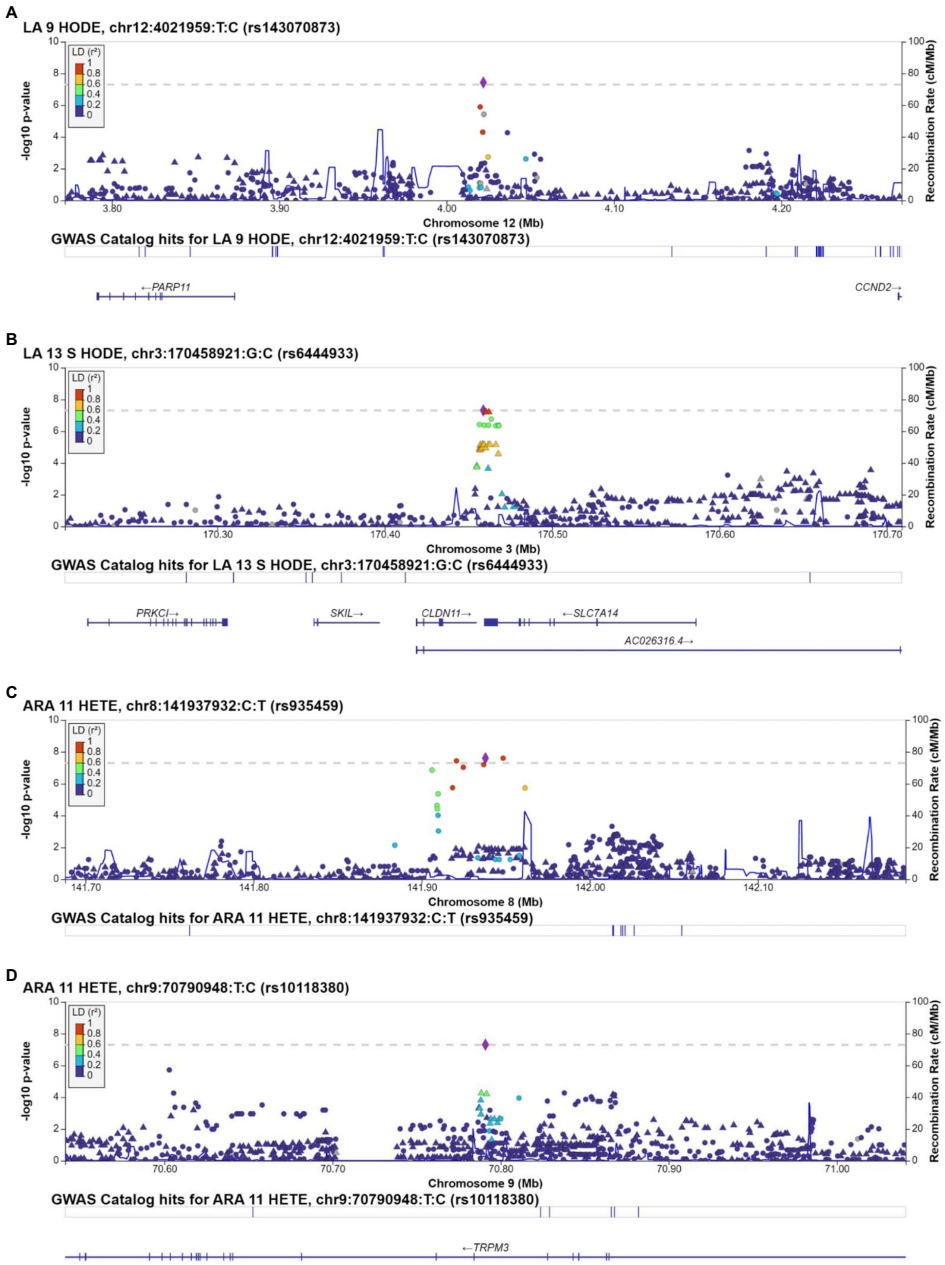
Regional plots for SNPs associated with individual oxylipins that met genome-wide significance (value of p<5 × 10^−8^). Regional plots are for rs143070873, associated with 9-HODE **(A)**; rs6444933, associated with 13 S-HODE **(B)**; rs935459, associated with 11-HETE **(C)**; and rs10118380, associated with 11-HETE **(D)**. Chromosome and physical location (Mb) are on the X-axis. Negative base ten logarithm of the *p*-values is on the left Y-axis, and recombination activity (cM/Mb) is shown on the right Y-axis as a blue line. Positions, recombination rates, and gene annotations are according to NCBI’s build 38 (hg 19) and the 1,000 Genomes Project Phase 3 multiethnic data set. LD is calculated for a European population. Plots display +/−500,000BP of the selected SNP.

We conducted GWAS using the top 5 loading oxylipins for oxylipin PC1, to further investigate the findings for oxylipin PC1. Manhattan plots are displayed in Supplementary Figures S4A–E. For the LA-related oxylipin 9-HODE, 1 SNP met genome-wide significance. The T allele for rs143070873, in *LOC105369611* on chromosome 12 was positively associated with 9-HODE (beta estimate: 2.26, *p* = 3.75 × 10^−8^) (Table 3). Flanking SNPs in LD exhibit similar levels of significance and direction of association (Figure 2A). There was 1 SNP that met genome-wide significance for 13 S-HODE which is synthesized from LA. The SNP rs6444933 located in the intergenic region between *CLDN11* and *SLC7A14* (beta estimate: −2.15, *p* = 4.96 × 10^−8^) was inversely associated with the 13 S-HODE. Several flanking SNPs in LD demonstrate similar levels of significance (Figure 2B). There were no SNPs that met genome-wide significance for the LA-related oxylipins 12,13-DiHOME and 9,10-DiHOME, or for the ALA-related oxylipin 9,10-DiHODE.

**Table 3:** Genome-wide significant SNPs associated with individual oxylipins.

SNP ID	rs number	Gene location	*β*	*p*	MAF	Rsq
*1 SNP met genome wide significance for 9-HODE (LA-related)*						
chr12:4021959:T:C	rs143070873	LOC105369611	2.26	3.75 × 10^−8^	0.05	0.89
*1 SNP met genome wide significance for 13 S-HODE (LA-related)*						
chr3:170458921:G:C	rs6444933	Between CLDN11 and SLC7A14	−2.15	4.96 × 10^−8^	0.38	0.98
*4 SNPs met genome wide significance for 11-HETE (ARA-related)*						
chr8:141920682:G:A	rs58388633	Between MIR1302-7 and LOC100131146	0.057	3.70 × 10^−8^	0.09	0.94
chr8:141937932:C:T	rs935459		0.057	2.50 × 10^−8^	0.09	0.94
chr8:141948453:T:C	rs67132476		0.057	2.50 × 10^−8^	0.09	0.96
chr9:70790948:T:C	rs10118380	TRPM3: Intron Variant	−0.034	4.91 × 10^−8^	0.47	0.99

GWAS were adjusted for sex and ancestry PC1-3.

SNPs are identified as chromosome number: base pair location: reference allele: alternate allele.

All beta estimates are calculated with respect to the reference allele (i.e., a positive beta estimate implies that the reference allele is associated with higher average levels of the corresponding oxylipin).

Minor allele frequency (MAF).

Rsq was calculated as the estimated squared correlation of the imputed genotype and the true (unobserved) genotype.

### 3.4. Oxylipin PC2

We investigated genetic determinants of an oxylipin profile characterized by ARA-related oxylipins (oxylipin PC2). No SNPs met genome-wide significance for oxylipin PC2 (value of *p*<5 × 10^−8^), and 255 SNPs met suggestive significance (value of *p*<5 × 10^−5^), adjusting for sex and ancestry (Figure 1D). We selected the two lead SNPs meeting suggestive significance criteria for regional plotting. The lead SNP rs972607, an intronic variant in *ITGA9* is illustrated in Figure 1E (beta estimate: 0.772, *p* = 7.20 × 10^−7^) (Table 2). SNPs downstream of this SNP in the *ITGA9* gene in LD with rs972607 exhibit similar levels of significance and positive beta estimates. The additional lead SNP, rs62027890, an intronic SNP in *LPCAT2* was positively associated with oxylipin PC2 (beta estimate: 0.618, *p* = 5.21 × 10^−7^) (Figure 1F).

### 3.5. Oxylipin PC2 individual oxylipins

We conducted GWAS of the top 5 loading oxylipins for oxylipin PC2 (Manhattan plots are displayed in Supplementary Figures S4F–J). We identified 4 SNPs (2 loci) associated with 11-HETE (an ARA-related oxylipin). Three SNPs located in the region on chromosome 8 between *MIR1302-7* and *LOC100131146* were associated with 11-HETE (Table 3). Several flanking SNPs in high LD exhibit similar levels of significance and directionality (Figure 2C). Secondly, the T allele of rs10118380, an intronic variant in *TRPM3*, was negatively associated with 11-HETE (beta estimate: −0.034, *p* = 4.91 × 10^−8^). SNPs in *TRPM3* in LD with rs10118380 demonstrate lower levels of significance and are also negatively associated with levels of 11-HETE (Figure 2D). There were no SNPs that met genome wide significance for 12 S-HETE, 5-HETE, 11,12-DiHETrE, or 14,15-DiHETrE, which were the other top 4 loading oxylipins from oxylipin PC2.

## 4. Discussion

To illuminate biologic pathways and potential therapeutic targets, we identified genetic predictors of oxylipins through a comprehensive GWAS of oxylipins and oxylipin profiles derived from PCA and selected based on clinical relevance identified in prior work. We identified 4 loci associated with the oxylipins 9-HODE, 13 S-HODE, or 11-HETE. No SNPs met genome-wide significance for the oxylipin profiles, but we identified supported peaks in the GWAS of oxylipin profiles that met suggestive significance. Loci associated with oxylipins and oxylipin profiles are involved in fatty acid metabolism (rs6092091, rs62027890), inflammation (rs6444933, rs10118380), and autoimmune conditions (rs972607).

We investigated genetic predictors of oxylipin PC1 through GWAS of the top 5 loading individual oxylipins on this PC. We found that rs6444933 was associated with 13-S-HODE, a LA-related oxylipin. This SNP is downstream of *CLDN11*, which encodes for claudin 11. *CLDN11* is upregulated by the inflammation-related cytokine IL-4 in macrophages (41). In the Framingham cohort, SNPs near *CLDN11* have also been found to interact with the PUFA oleic acid to predict levels of monocyte chemoattractant protein-1 (MCP-1), which participates in the inflammatory cascade (42) further demonstrating the relationship between inflammation and *CLDN11* and potential involvement of PUFA. Claudin-11 is also a well-established target antigen of the autoimmune condition multiple sclerosis (43, 44).

Although there were no SNPs that met genome-wide significance for oxylipin PC1, we investigated the top two SNPs through regional plotting. The SNP rs6092091 is located in an intergenic region between *TGM2* and *KIAA1755* and was flanked by several SNPs in LD with suggestive levels of significance, indicating this region may play a role in an oxylipin profile characterized by LA- and ALA-related oxylipins. Interestingly, *TGM2* may increase the enzyme activity of PLA2, which cleaves fatty acids phospholipids, an initial step in oxylipin biosynthesis, and may increase eicosanoid synthesis in airway epithelial cells, increasing inflammation (45). This interplay between *TGM2* and *PLA2* may explain this suggestive association. We have found that oxylipins are associated with risk of the autoimmune disease T1D (13), and *TGM2* encodes for the protein transglutaminase 2, a target of autoantibodies in another autoimmune condition, celiac disease (46). rs6092091 is also downstream of *KIAA1755*, which has been established as a heart rate locus (47). *KIAA1755* was also identified through loss of function analysis to potentially impact levels of EPA (48). Although oxylipin PC1 was not strongly characterized by EPA-related oxylipins, it did represent higher levels of ALA-related oxylipins, another n3 fatty acid. This provided further evidence that loci associated with oxylipin levels are related to inflammation and autoimmune conditions.

We also conducted GWAS of the top loading oxylipins for oxylipin PC2 to explore genetic predictors of ARA-related oxylipins. Two loci were associated with the ARA-related oxylipin 11-HETE, including rs10118380, an intronic variant in *TRPM3* (Transient Receptor Potential Cation Channel Subfamily M Member 3), which is upregulated under pro-inflammatory conditions (49, 50). Particularly, *TRPM3* is involved with pain sensations during inflammation (51). Further, other transient receptor potential channels are signaled *via* oxylipins through inflammatory and pain cascades (52–55). *TRPM3* has also been shown to impact insulin secretion by beta cells (56, 57). Indeed, a variant in *TRPM3* significantly interacted with fruit and vegetable intake in European-ancestry individuals and with intake of cooked vegetable in East Asian-ancestry individuals to predict hemoglobin A1C levels (58). With our previous findings on oxylipins and T1D (13), this supports a complex relationship between *TRPM3*, oxylipins, and insulin secretion.

We explored the two lead SNPs for oxylipin PC2, which represented ARA-related oxylipins, although these SNPs did not meet genome wide significance. The SNP rs62027890 is an intronic variant for *LPCAT2*, which encodes for lysophosphatidylcholine acyltransferase 2. *LPCAT2* is induced through pro-inflammatory cytokines (59). Importantly, *LPCAT2* is highly expressed in macrophages and neutrophils, both inflammatory cells. *LPCAT2* participates in incorporating FA into the phospholipid membrane, particularly ARA and may be co-regulated with *PLA2* (60). This relationship between *LPCAT2* and ARA-related oxylipins is reinforced with the findings that gene expression of Lpcat2 in mice was positively associated with n6 oxylipins, and negatively with n3 oxylipins (61). Finally, Wolf et al. found that *LPCAT3*, another lysophosphatidylcholine acyltransferase was associated with a PC of n3 and n6 FA (21). *LPCAT2* may play an important role in the generation of ARA-related oxylipins. The SNP rs972607 also met suggestive significance for oxylipin PC2 and is an intronic variant in the gene *ITGA9,* encoding alpha integrin. *ITGA9* plays a role in inflammation (62) as well as the autoimmune diseases rheumatoid arthritis (63, 64) and autoimmune encephalomyelitis (65) in animal models.

It is interesting that the genome-wide significant hits are only from the individual oxylipin analyses. This suggests that genetic variants may have oxylipin-specific effects, rather than a single SNP that influences multiple oxylipins. Although studying multiple oxylipins together was more clinically relevant in our analyses in T1D (13), when testing specific mechanisms under genetic control, individual oxylipins may be more pertinent. The oxylipin PCs also include oxylipins that may not be heavily influenced by genetic factors (e.g., 12 S-HETE, 11,12-DiHETrE, or 14,15-DiHETrE), and this noise may have made it difficult to capture a significant genetic signal. Although there was no direct overlap between the genome-wide significant hit for oxylipin PC2 and the other SNPs we identified, there is a shared interaction with *PLA2* and involvement in inflammation and autoimmunity, as noted above. The oxylipin profiles developed for our study were derived from the data-driven approach of PCA, rather than the development of profiles based on specific substrates or enzymes. While examination of the loading values of our PCs did reveal patterns based on substrate (i.e., oxylipin PC1 represented largely LA- and ALA-related oxylipins, and oxylipin PC2 represented largely ARA-related oxylipins), we acknowledge that a more intentional identification of profiles based on substrate or enzymes might reveal additional genetic associations.

As noted, Bermingham et al. found that genetic and environmental predictors both contribute to oxylipin variation, and the extent to which genetic factors contribute varied by oxylipin (27). Indeed, the results of our GWAS suggest that genetic control of oxylipin levels may vary by the oxylipin. The loci identified in our GWAS may provide opportunities for therapeutic targets in autoimmune and inflammatory conditions. Additionally, oxylipins under less genetic control may be more susceptible to modifiable environmental or behavioral factors. Both genetic and environmental factors are important to consider in the study of oxylipins and disease.

Strengths of this study include measurement of multiple oxylipins at multiple time points. The use of a summary measure of these multiple time points for the oxylipins allows for a more stable estimate of oxylipin levels. The use of PCA captured biologically relevant oxylipin profiles (relevant based on precursor FA and association with T1D). Weaknesses include the small sample size that limits power to detect variants. This study was drawn from a population of children at risk of T1D that mostly reported NHW ethnicity, which limits generalizability. The samples for this study were non-fasting, which may add noise to oxylipin levels (66, 67), although this noise would be non-differential and would bias our findings toward the null. A limited number of oxylipins derived from EPA and DHA were measured, and did not contribute largely to the oxylipin profile, so we were not able to comprehensively investigate genetic variants related to EPA- and DHA- derived oxylipins.

### 4.1. Conclusion

We identified loci associated with the LA-related oxylipins 9-HODE and 13 S-HODE, as well as the ARA-related oxylipin 11-HETE. We also identified suggestive supported peaks associated with oxylipin PC1 and PC2. These loci demonstrate complex interaction with inflammatory pathways and autoimmune diseases with oxylipins. Additional studies are needed to replicate these findings and investigate genetic determinants of individual oxylipins, including those derived from EPA and DHA. Future studies on the causal role of oxylipins in the development of these autoimmune diseases will contribute to our understanding of these genetic loci as potential targets for autoimmune and inflammation-related conditions.

## Data availability statement

The original contributions presented in the study are included in the article/Supplementary material, further inquiries can be directed to the corresponding author.

## Ethics statement

The studies involving human participants were reviewed and approved by Colorado Multiple Institutional Review Board. Written informed consent to participate in this study was provided by the participants' legal guardian/next of kin.

## Author contributions

TB designed the study, performed data analysis, interpreted the data, and drafted the manuscript. JN designed the study, interpreted the data, and edited the manuscript. OF, SO-G, and W-MC collected data and reviewed and edited the manuscript. RJ, LV, PC, AR, SK, BF, TC, WP, KK, and MR reviewed and edited the manuscript. All authors contributed to the article and approved the submitted version.

## Funding

The study was performed at the Barbara Davis Center for Childhood Diabetes in Denver, CO. This work was supported by the National Institutes of Health R01-DK104351 and R01- DK32493. Supported was also provided by NIH/NCATS Colorado CTSA grant number TL1 TR002533, the University of Colorado Diabetes Research Center (P30-DK116073) the Colorado Clinical and Translational Sciences Institute (KL2-TR002534) and the American Diabetes Association (ADA-7-22-ICTSPM-08).

## Conflict of interest

The authors declare that the research was conducted in the absence of any commercial or financial relationships that could be construed as a potential conflict of interest.

The reviewer NL declared a shared affiliation with OF to the handling editor at the time of review.

## Publisher’s note

All claims expressed in this article are solely those of the authors and do not necessarily represent those of their affiliated organizations, or those of the publisher, the editors and the reviewers. Any product that may be evaluated in this article, or claim that may be made by its manufacturer, is not guaranteed or endorsed by the publisher.
