# Supplementary material for: Genome-wide analysis of oxylipins and oxylipin profiles in a pediatric population
Source: Front Nutr. 2023 Mar 28;10:1040993. doi: 10.3389/fnut.2023.1040993 (PMC10086335; doi:10.3389/fnut.2023.1040993)
Supplement: Supplementary file 1 [file Data_Sheet_1.docx]

Supplementary Material

# Supplementary Figures and Tables

| **Supplemental Table S1: Oxylipin Full Names** | | | |
| --- | --- | --- | --- |
| **Abbreviation** | **Full Name** | **Precursor** | **Enzyme** |
| **n6** | | | |
| 9(10)-EpOME | 9(10)-Epoxy-12Z-octadecenoic acid | LA | CYP450 |
| 9,10-DiHOME | 9,10-Dihydroxyoctadec-12-enoic acid | LA | CYP450 sEH |
| 9,12,13-TriHOME | 9,12,13-Trihydroxyoctadec-10-enoic acid | LA | 12/15-LOX |
| 9-HODE | 9-Hydroxylinoleic acid | LA | 5-LOX, non-enzymatic |
| 9-KODE | 9-Ketooctadeca-10,12-dienoic acid | LA | 5-LOX, non-enzymatic |
| 12(13)-EpOME | 12,13-Epoxy-9-octadecenoic acid | LA | CYP450 |
| 12,13-DiHOME | 12,13-Dihydroxyoctadec-9-enoic acid | LA | CYP450 sEH |
| 13S-HODE | 13-Hydroxyoctadecadienoic acid | LA | 12/15-LOX, non-enzymatic |
| 5-HETE | 5-Hydroxy-6,8,11,14-eicosatetraenoic acid | ARA | 5-LOX |
| 5,6-DiHETrE | 5,6-Dihydroxyeicosa-8,11,14-trienoic acid | ARA | CYP450 sEH |
| 8,9-DiHETrE | 8,9-Dihydroxyeicosa-5,11,14-trienoic acid | ARA | CYP450 |
| 8S-HETE | 8-Hydroxyeicosa-5,9,11,14-tetraenoic acid | ARA | 12/15-LOX |
| 9-HETE | 9-Hydroxyeicosa-5,7,11,14-tetraenoic acid | ARA | 12/15-LOX, non-enzymatic |
| 11,12-DiHETrE | 11,12-Dihydroxyicosa-5,8,14-trienoic acid | ARA | CYP450 sEH |
| 11-HETE | 11-Hydroxy-arachidonic acid | ARA | 12/15-LOX, COX, non-enzymatically |
| 12S-HETE | 12-Hydroxy-5,8,10,14-eicosatetraenoic acid | ARA | 12/15-LOX |
| 14,15-DiHETrE | 14,15-Dihydroxyeicosa-5,8,11-trienoic acid | ARA | CYP450 sEH |
| 15-HETE | 15-Hydroxyeicosa-5,8,11,13-tetraenoic acid | ARA | 12/15-LOX, COX |
| 15-KETE | 15-Ketoeicosa-5,8,11,13-tetraenoic acid | ARA | 12/15-LOX |
| Lipoxin A4 | 5,6,15-Trihydroxyeicosa-7,9,11,13-tetraenoic acid | ARA | 12/15-LOX |
| LTB4 | Leukotriene B4 | ARA | 5-LOX |
| PGF2α | Prostaglandin F2a | ARA | COX |
| **n3** | | | |
| 9-HOTE | 9-Hydroxyoctadeca-10,12,15-trienoic acid | ALA | COX, non-enzymatic |
| α-9(10)-EpODE | 9(10)-Epoxy-12,15-octadecadienoic acid | ALA | CYP450 |
| 9,10-DiHODE | 9,10-Dihydroxyoctadeca-12,15-dienoic acid | ALA | CYP450 |
| α-12(13)-EpODE | 12(13)-Epoxy-9,15-octadecadienoic acid | ALA | CYP450 |
| 12,13-DiHODE | 12,13-Dihydroxyoctadeca-9,15-dienoic acid | ALA | CYP450 sEH |
| 13-HOTE | 13-Hydroxyoctadeca-9,11,15-trienoic acid | ALA | COX, non-enzymatic |
| 15,16-EpODE | 15(16)-Epoxy-9,12-octadecadienoic acid | ALA | CYP450 |
| 15,16-DiHODE | 15,16-Dihydroxyoctadeca-9,12-dienoic acid | ALA | CYP450 sEH |
| 4-HDoHE | 4-Hydroxydocosa-5,7,10,13,16,19-hexaenoic acid | DHA | 12/15-LOX |
| 14-HDoHE | 14-Hydroxydocosa-4,7,10,12,16,19-hexaenoic acid | DHA | 12/15-LOX, non-enzymatic |
| 17-HDoHE | 17-Hydroxy-4,7,10,13,15,19-docosahexaenoic acid | DHA | 12/15-LOX, non-enzymatic |
| 19,20-DiHDPE | 19,20-Dihydroxydocosa-4,7,10,13,16-pentaenoic acid | DHA | CYP450 sEH |
| 15-HEPE | 15-Hydroxyeicosa-5,8,11,13,17-pentaenoic acid | EPA | 12/15-LOX |
| 17,18-DiHETE | 17,18-Dihydroxyeicosa-5,8,11,14-tetraenoic acid | EPA | CYP450 sEH |
| Linoleic Acid (LA), Arachidonic Acid (ARA), Alpha-Linolenic Acid (ALA), Docosahexaenoic acid (DHA), Eicosapentaenoic acid (EPA)  Cytochrome P450 (CYP450), Soluble Epoxide Hydrolase (sEH), Cyclooxygenase (COX), Lipoxygenases (LOX) | | | |

**Supplemental Figure S1:**


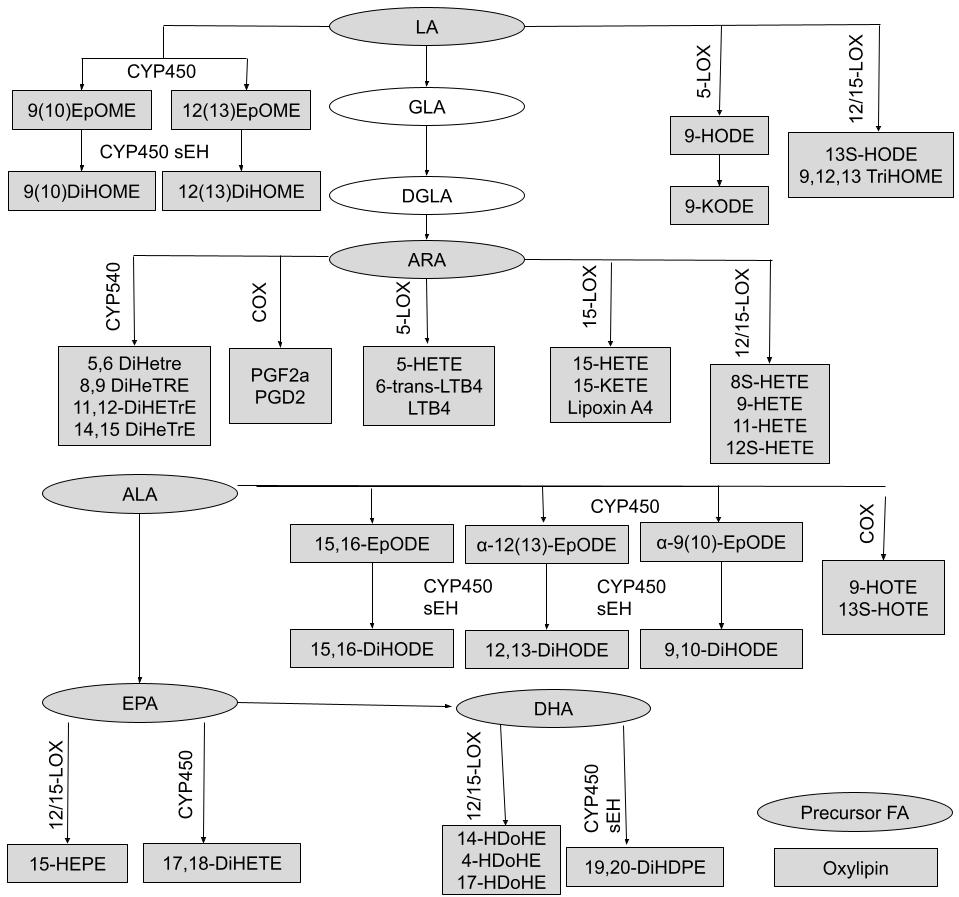


**Supplemental Figure S1**: Relationships between precursor FA and oxylipins. Boxes represent oxylipins and ovals represent precursor FA. Shaded boxes represent metabolites that were measured by this study, and unshaded boxes represent unmeasured metabolites. Biosynthetic enzymes for the formation of oxylipins (CYP450 sEH, 12/15-LOX, 5-LOX, 15-LOX, COX) are indicated above the oxylipin formed by the enzyme. Full names of oxylipins are presented in Supplemental Table 1.

| **Supplemental Table S2: PCA Loadings of Average Oxylipin Levels** | | | |
| --- | --- | --- | --- |
| Oxylipin | Precursor FA | **PC1 loading**  **Eigenvalue:**  9.961 | **PC2 loading**  **Eigenvalue:**  4.404 |
| 9(10)-EpOME | LA | **72** | -7 |
| 9,10-DiHOME | LA | **83** | -15 |
| 9,12,13-TriHOME | LA | **33** | -13 |
| 9-HODE | LA | **84** | 0 |
| 9-KODE | LA | **73** | 7 |
| 12(13)-EpOME | LA | **81** | -23 |
| 12,13-DiHOME | LA | **86** | -12 |
| 13-HOTE | LA | **75** | -4 |
| 13S-HODE | LA | **85** | -10 |
| 5-HETE | ARA | 0 | **71** |
| 5,6-DiHETrE | ARA | 16 | **60** |
| 8,9-DiHETrE | ARA | 11 | **39** |
| 8S-HETE | ARA | -3 | **40** |
| 9-HETE | ARA | 4 | **49** |
| 11,12-DiHETrE | ARA | **35** | **65** |
| 11-HETE | ARA | 4 | **76** |
| 12S-HETE | ARA | 8 | **71** |
| 14,15-DiHETrE | ARA | **38** | **63** |
| 15-HETE | ARA | 1 | **58** |
| 15-KETE | ARA | 0 | -3 |
| Lipoxin A4 | ARA | -8 | -7 |
| LTB4 | ARA | 4 | 12 |
| PGF2alpha | ARA | 3 | 11 |
| Alpha-9(10)-EpODE | ALA | **79** | -2 |
| 9,10-DiHODE | ALA | **83** | -16 |
| alpha-12(13)-EpODE | ALA | **76** | -20 |
| 12,13-DiHODE | ALA | **74** | -5 |
| 15,16-EpODE | ALA | **80** | 0 |
| 15,16-DiHODE | ALA | **74** | -24 |
| 9-HOTE | ALA | **70** | 14 |
| 4-HDoHE | DHA | -5 | 7 |
| 14-HDoHE | DHA | 6 | **45** |
| 17-HDoHE | DHA | 8 | 5 |
| 19,20-DiHDPE | DHA | **42** | **37** |
| 15-HEPE | EPA | 7 | 10 |
| 17,18-DiHETE | EPA | **29** | **28** |
| Subject-specific intercepts of 36 oxylipins were used in a Principal Components Analysis (PCA). Based on scree plot, the first two principal components (PCs) were extracted.  Loading values greater than ±0.277501 (the root mean square of all loading values in the PCA) are flagged (see **bold** font). For ease of presentation, loadings are multiplied by 100 and rounded to the nearest integer. | | | |

**Supplemental Figure S2:**

 
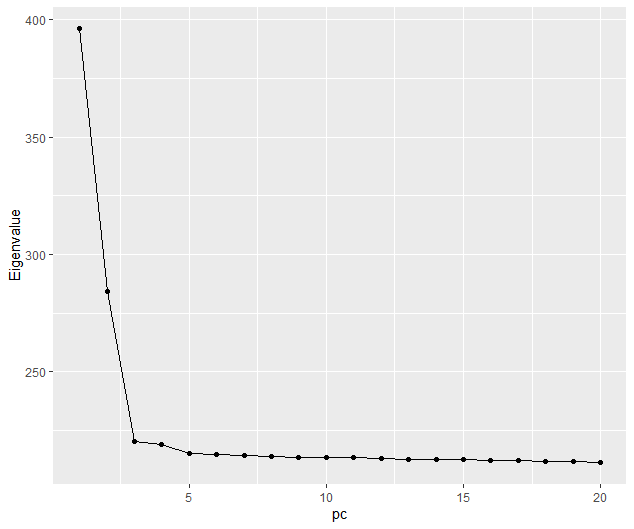


**Supplemental Figure S2**: Scree plot of ancestry Principal Components Analysis

**Supplemental Figure S3: Histogram of p-values for oxylipin PC1, PC2, and individual oxylipins**

A

B
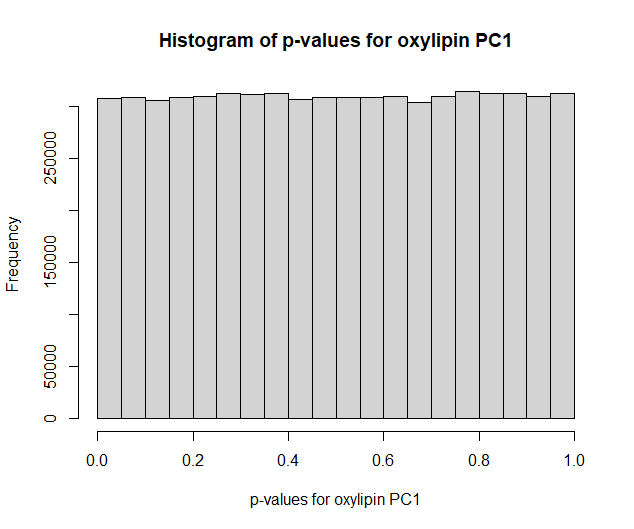


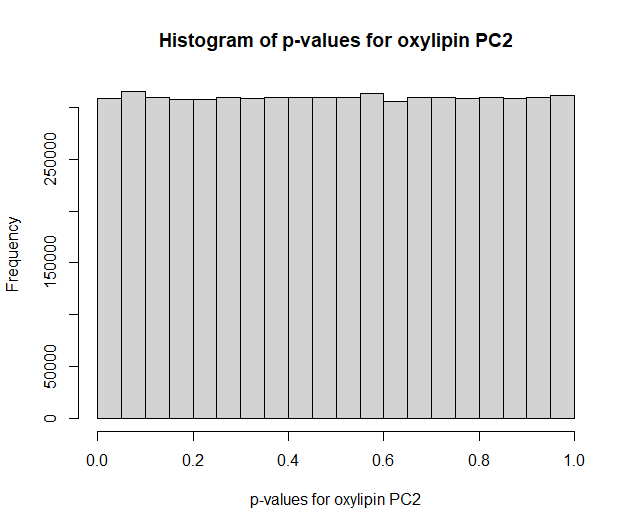


C

D
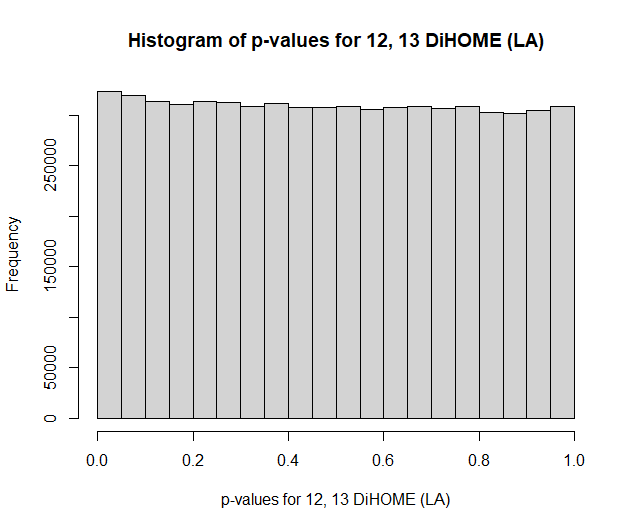


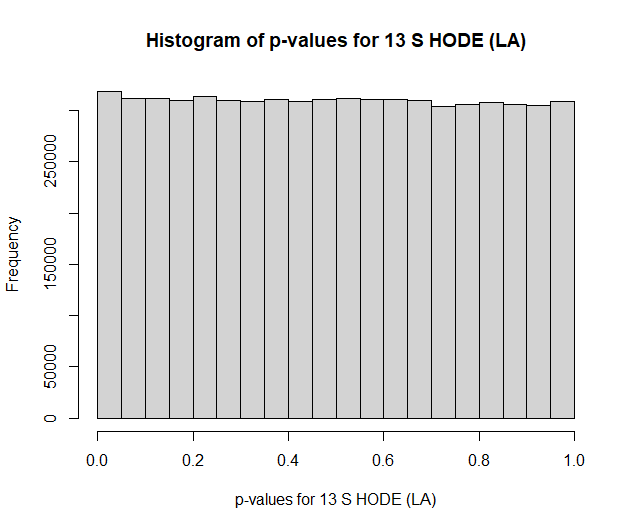


E

F
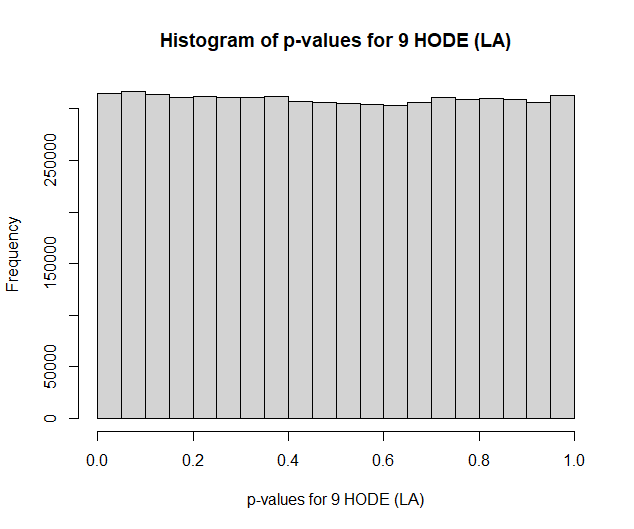

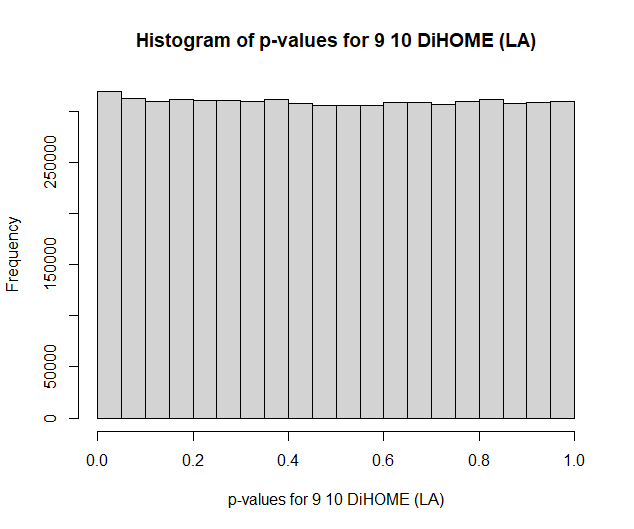


G

H
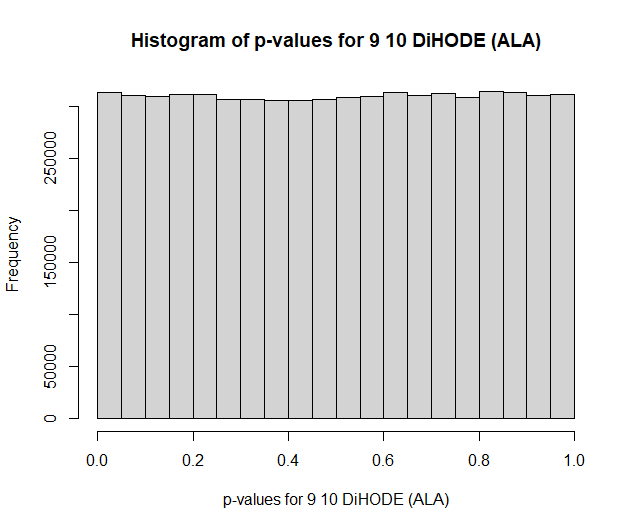

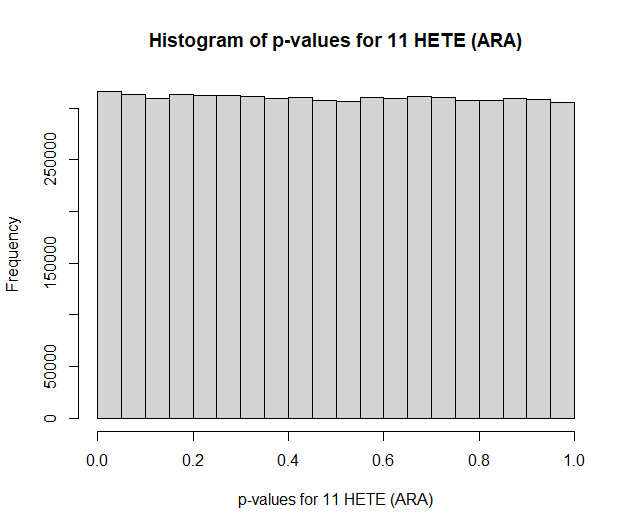


I

J
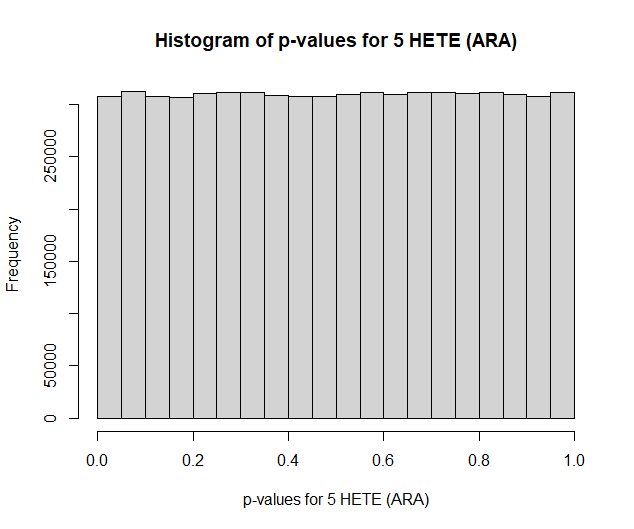

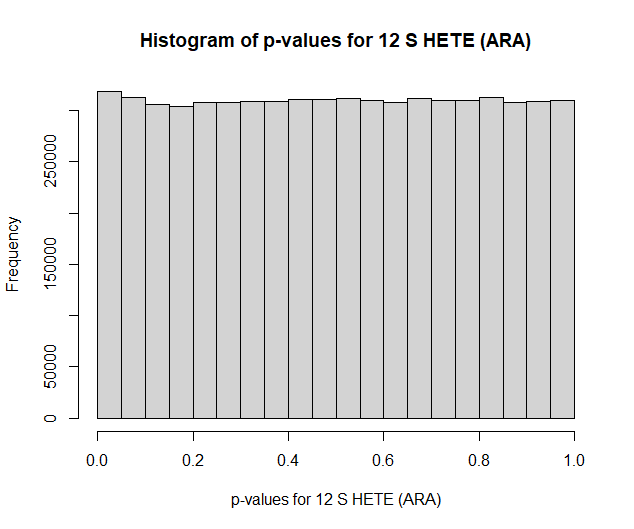


K

L
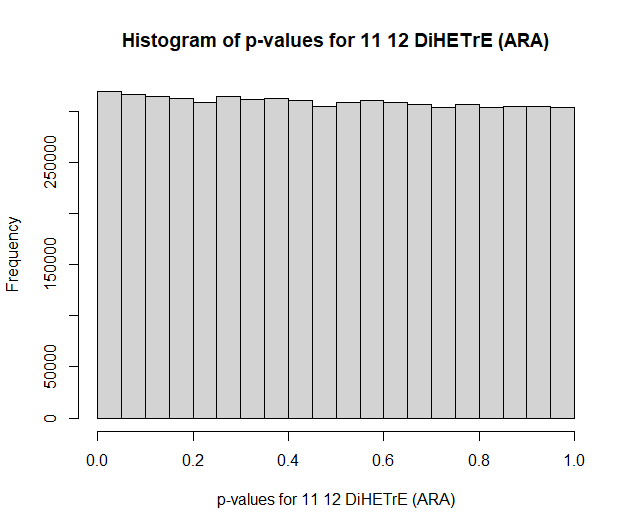

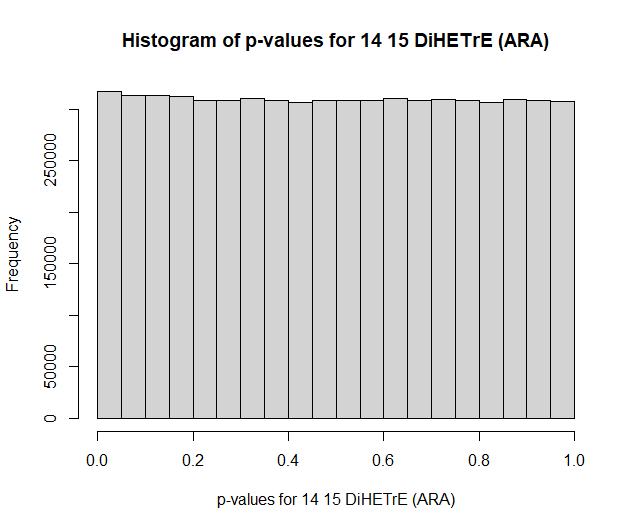


**Supplemental Figure S3:** Distribution of p-values from genome-wide analysis study (GWAS) of Oxylipin PC1 (A), Oxylipin PC2 (B), 12, 13-DiHOME (C), 13 S-HODE (D), 9-HODE (E), 9, 10-DiHOME (F), 9, 10-DiHODE (G), 11-HETE (H), 5-HETE (I), 12 S-HETE (J), 11,12-DiHETrE (K), 14,15-DiHETrE (L).

**Supplemental Figure S4: Manhattan plot for individual oxylipins**

A


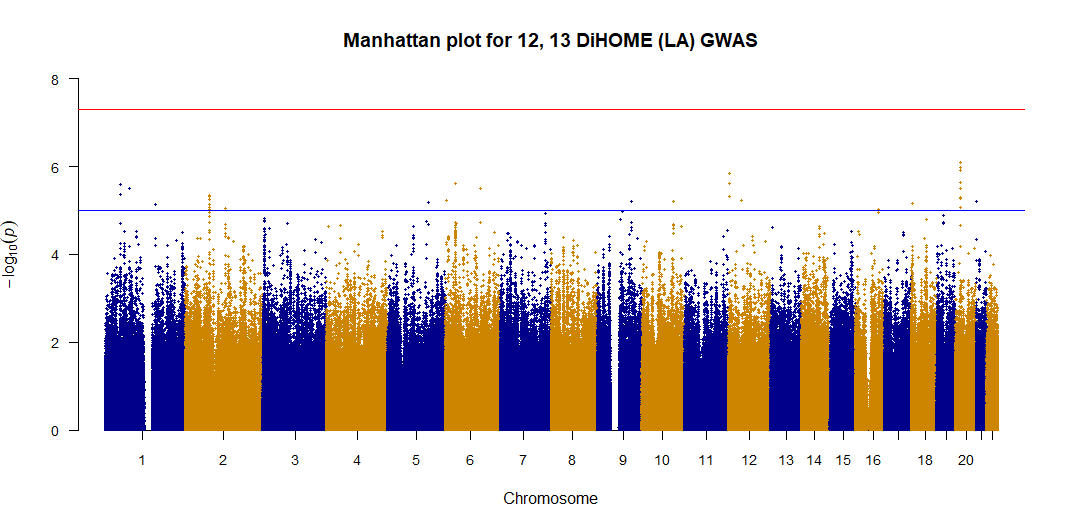


B


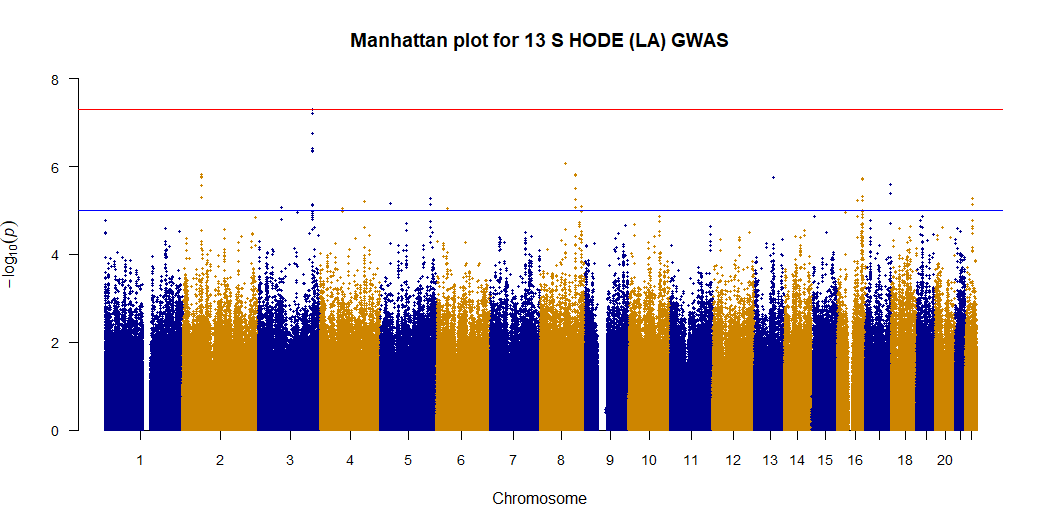


C


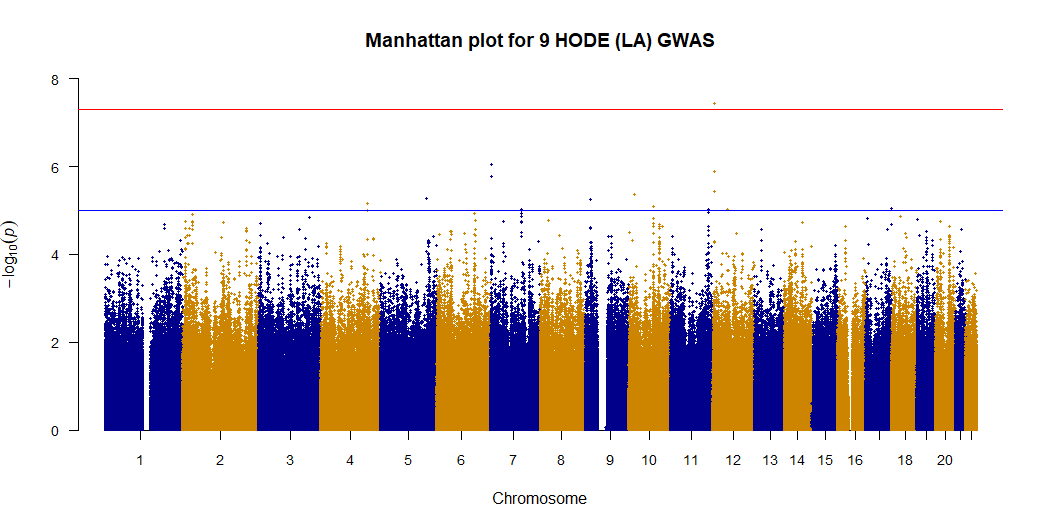


D


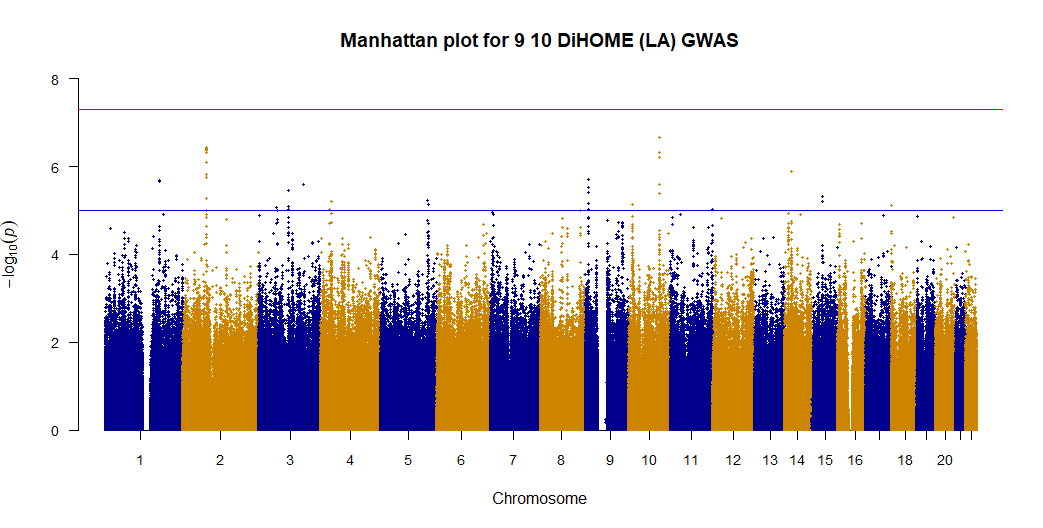


E


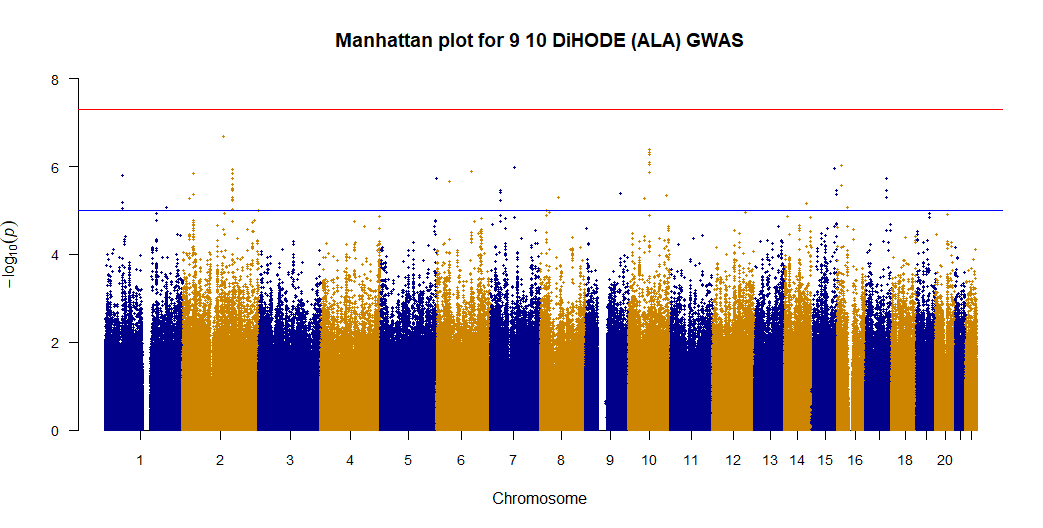


F


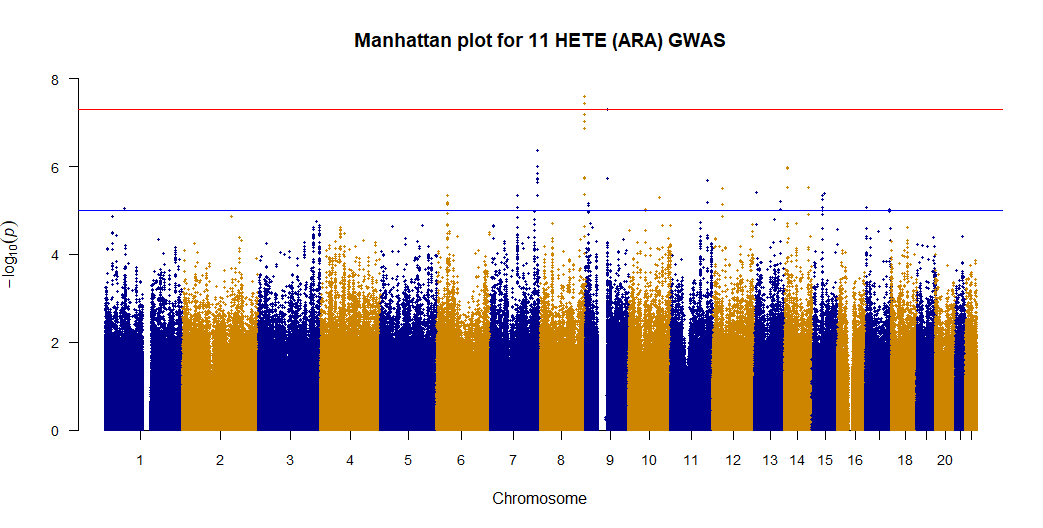


G


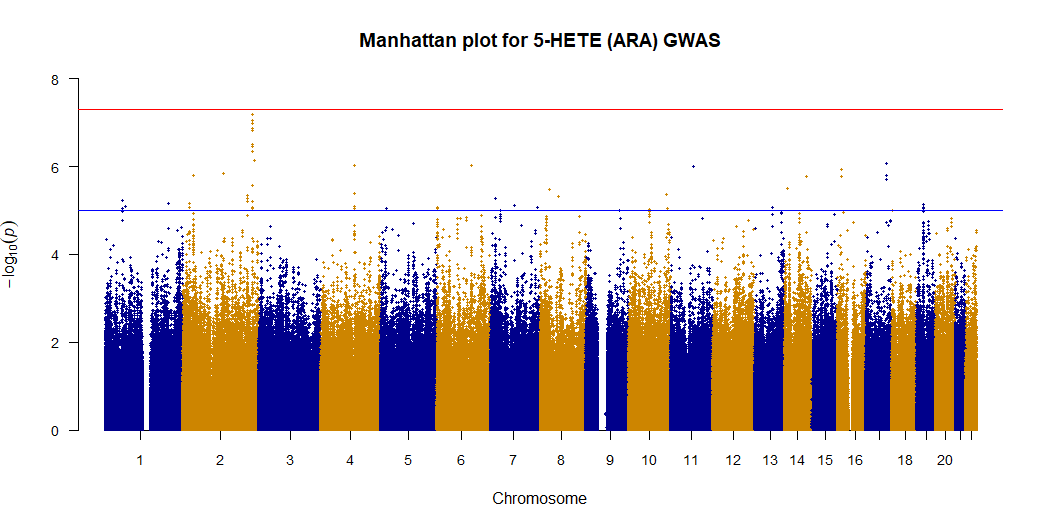


H


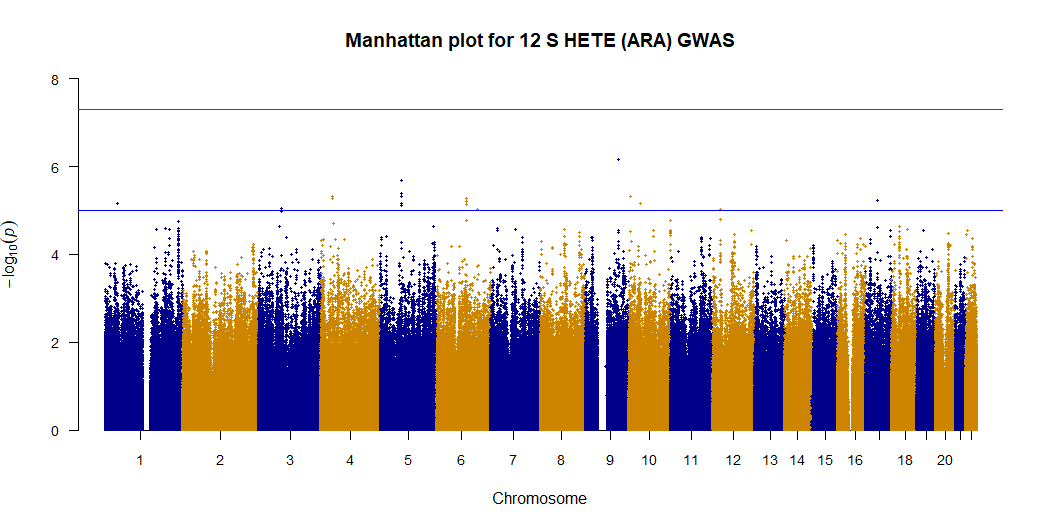


I


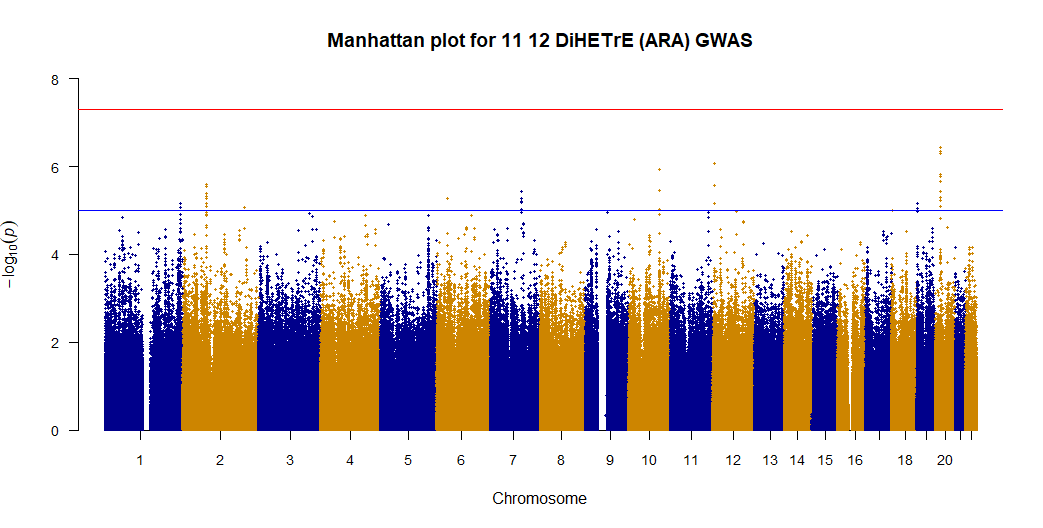


J


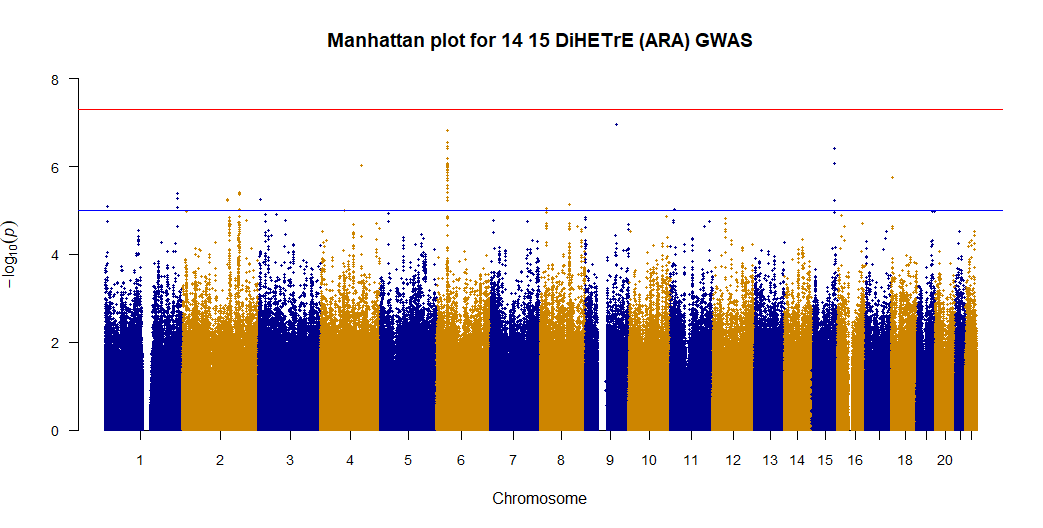


**Supplemental Figure S4**: Genome-wide association study (GWAS) results of top loading oxylipins from oxylipin PC1 and PC2, adjusted for sex and ancestry. From Oxylipin PC1, we conducted GWAS with 12,13-DiHOME (LA) (A), 13 S-HODE (LA) (B), 9 HODE (LA) (C), 9,10-DiHOME (LA) (D), and 9,10-DiHODE (ALA) (E). From Oxylipin PC2, we conducted GWAS with 11-HETE (ARA) (F), 5-HETE (ARA) (G), 12 S-HETE (ARA) (H), 11,12 DiHETrE (ARA) (I), and 14,15-DiHETrE (ARA) (J). Suggestive level of significance is p-value<5x10^-5^ (blue line), and genome-wide significance is p-value<5x10^-8^ (red line).
